# Supplementary figures and images for: Clinical features and severe acute respiratory syndrome-coronavirus-2 structural protein-based serology of Mexican children and adolescents with coronavirus disease 2019
Source: PLoS One. 2022 Aug 15;17(8):e0273097. doi: 10.1371/journal.pone.0273097 (PMC9377623; doi:10.1371/journal.pone.0273097)

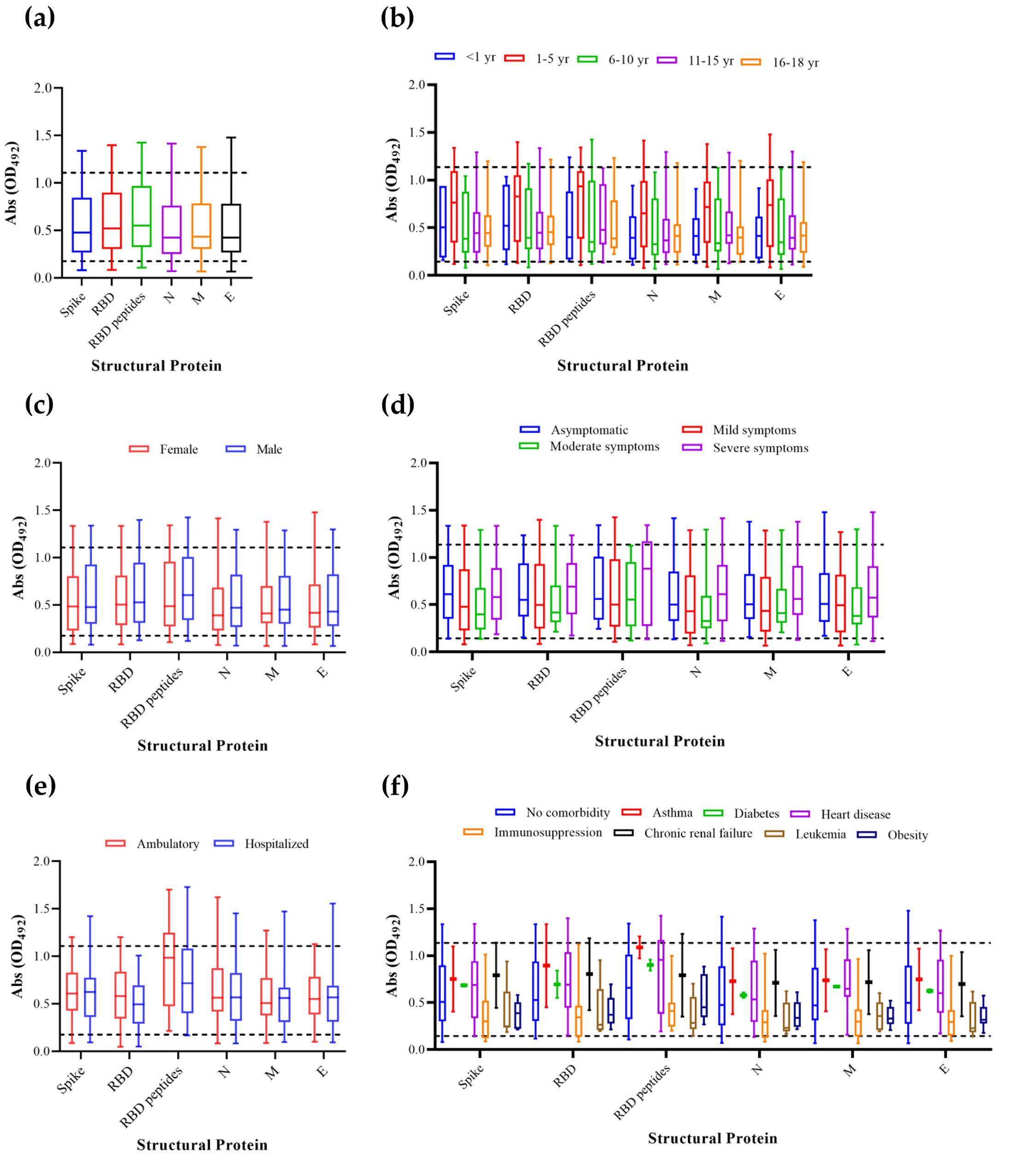

Supplement: S1 Fig — IgG determination of gamma chain IgG antibodies was performed by ELISA in (a) total IgG antibodies, comparisons between IgG and (b) age, (c) gender, (d) severity of illness, (e) patient status, and (f) comorbidity. Bars represent the median of 100 determinations from the sera of children and adolescents with COVID-19 performed in duplicate. Top line represents the median value of 10 COVID-19-positive patients, and bottom line indicates the median value of 10 COVID-19-negative volunteers. Statistical significance was determined by ANOVA followed by the Kruskal–Wallis post hoc test. Statistical significance was considered when *p≤ 0.05 to >0.01, **≤0.01 to >0.002, ***≤0.001 to >0.0001, and ***≤0.0001. (TIF) [file pone.0273097.s001.tif]
