# Supplementary material for: Clinical features and severe acute respiratory syndrome-coronavirus-2 structural protein-based serology of Mexican children and adolescents with coronavirus disease 2019
Source: PLoS One. 2022 Aug 15;17(8):e0273097. doi: 10.1371/journal.pone.0273097 (PMC9377623; doi:10.1371/journal.pone.0273097)
Supplement: Attachment — Submitted filename: Response to Reviewers.docx [file pone.0273097.s002.docx]

**Response to Reviewers**

We really appreciate the editor´s and reviewer´s suggestions made to the manuscript. Below we provide the point-by-point response to comments. We strongly believe that these modifications have improved the manuscript.

***Journal Requirements:***

When submitting your revision, we need you to address these additional requirements.

1. Please ensure that your manuscript meets PLOS ONE's style requirements, including those for file naming. The PLOS ONE style templates can be found at

https://journals.plos.org/plosone/s/file?id=wjVg/PLOSOne_formatting_sample_main_body.pdf and

<https://journals.plos.org/plosone/s/file?id=ba62/PLOSOne_formatting_sample_title_authors_affiliations.pdf>

**R1. We revised the manuscript based on PLOS ONE's style requirements and PLOS ONE style templates.**

2. Please provide additional details regarding participant consent. In the ethics statement in the Methods and online submission information, please ensure that you have specified what type you obtained (for instance, written or verbal, and if verbal, how it was documented and witnessed). If your study included minors, state whether you obtained consent from parents or guardians. If the need for consent was waived by the ethics committee, please include this information.

**R2. In this study, the parents accepted in written format the use of blood samples by signing the informed consent and settlement letters and the names of the patients were anonymized. In addition, the format of the letter was submitted to the database repository (https://doi.org/10.7910/DVN/DQNW2J) and the sentence was rewritten on page 7 and lines 166-169.**

3. We note that you have indicated that data from this study are available upon request. PLOS only allows data to be available upon request if there are legal or ethical restrictions on sharing data publicly. For more information on unacceptable data access restrictions, please see <http://journals.plos.org/plosone/s/data-availability#loc-unacceptable-data-access-restrictions>.

**R3a. The individual patient data used to generate the findings of this study are available on Harvard Dataverse (**[**https://dataverse.harvard.edu**](https://dataverse.harvard.edu)**) upon acceptance of the manuscript. In addition, the format of the consent and settlement letters was included in this data repository. DOI access is https://doi.org/10.7910/DVN/DQNW2J**

In your revised cover letter, please address the following prompts:

a) If there are ethical or legal restrictions on sharing a de-identified data set, please explain them in detail (e.g., data contain potentially sensitive information, data are owned by a third-party organization, etc.) and who has imposed them (e.g., an ethics committee). Please also provide contact information for a data access committee, ethics committee, or other institutional body to which data requests may be sent.

b) If there are no restrictions, please upload the minimal anonymized data set necessary to replicate your study findings as either Supporting Information files or to a stable, public repository and provide us with the relevant URLs, DOIs, or accession numbers. For a list of acceptable repositories, please see http://journals.plos.org/plosone/s/data-availability#loc-recommended-repositories.

We will update your Data Availability statement on your behalf to reflect the information you provide.

**R3b. The cover letter was revised and rewritten following their comments**

4. We note that you have included the phrase “data not shown” in your manuscript. Unfortunately, this does not meet our data sharing requirements. PLOS does not permit references to inaccessible data. We require that authors provide all relevant data within the paper, Supporting Information files, or in an acceptable, public repository. Please add a citation to support this phrase or upload the data that corresponds with these findings to a stable repository (such as Figshare or Dryad) and provide and URLs, DOIs, or accession numbers that may be used to access these data. Or, if the data are not a core part of the research being presented in your study, we ask that you remove the phrase that refers to these data.

**R4. The data are not a core part of the research in this study, we have removed the phrase that refers to these data on page 12 and line 279.**

5. Please review your reference list to ensure that it is complete and correct. If you have cited papers that have been retracted, please include the rationale for doing so in the manuscript text, or remove these references and replace them with relevant current references. Any changes to the reference list should be mentioned in the rebuttal letter that accompanies your revised manuscript. If you need to cite a retracted article, indicate the article’s retracted status in the References list and also include a citation and full reference for the retraction notice.

**R5. The reference list was revised and only was identified error in printing or writing on three papers. The changes were made with track changes on the manuscript.**

***Additional Editor Comments (if provided):***

Considering the opinion of the independent reviewer, it would be convenient to discuss why the analysis of neutralizing antibodies was not included
IS A WELL-STRUCTURED ARTICLE WITH A WELL-PLANNED EXPERIMENTAL DESIGN THAT ONLY NEEDS A CAREFUL REVIEW OF THE USE OF LANGUAGE,

**Reply to editor comments. We have edited the manuscript for language, grammar, structure, and content, from the aspect of fluency and nativity. In addition, we submit the edition certificate from ENAGO Academy.**

***Reviewers' comments:***

Reviewer's Responses to Questions

***Comments to the Author***

5. Review Comments to the Author

Reviewer #1: Nice study addressing the gap of knowledge on antibody responses of children. It would be useful if you could comment on level of correlation of different assays. Also why the neutralization assays were not performed at least to confirm few samples since all the assays are ELISA based.

**Reply to reviewer 1.**

**The aim of this study was to analyze the clinical features and IgG antibodies response against SARS-CoV-2 structural proteins of children and adolescents who visited the Hospital Infantil de México Federico Gómez. The evaluation of neutralizing antibodies in sera sample from SARS-CoV-2 infected patients is part of another study. We want to share part of these studies. Briefly, we have evaluated our MAP8 peptides-based ELISA method for infected SARS-CoV-2, which is under review in Scientific Reports (Cortés-Sarabia et al., SREP-22-00486A). In other study, we have compared the different in-home and commercial (validated) methodologies of S and N proteins based-ELISA (including MAP8 peptides) for determination of IgG antibodies from infected SARS-CoV-2 and vaccinated against COVID-19 sera. In addition, in-home and commercial methods for determination of neutralizing antibodies were correlated with all serology methods using RBD-HRP and soluble hACE2-Fc-based ELISA and viral pseudo particles (VSVG-luc-SARS-CoV-2-S)-based method. These findings showed that the in-house ELISA methods (MAP8 peptides, RBD, and S protein) used in our submitted manuscript have a high correlation with methods for determination of neutralizing antibodies (manuscript in preparation). The SARS-CoV-2 structural proteins (S, N, M and E)-based ELISA method is being also evaluated in a retrospective study using several sera from 2003, 2010 and 2020 to date for validation of our methods (In process).**
